# Supplementary material for: Codes between Poles: Linking Transcriptomic Insights into the Neurobiology of Bipolar Disorder
Source: Biology (Basel). 2024 Sep 30;13(10):787. doi: 10.3390/biology13100787 (PMC11505342; doi:10.3390/biology13100787)
Supplement: Supplementary file 1 [file biology-13-00787-s001.zip › biology-3187776-supplementary.pdf]

SUPPLEMENTARY MATERIAL

Codes Between Poles: Linking Transcriptomic Insights into the Neurobiology of Bipolar Disorder

Jon Patrick T. Garcia and Lemmuel L. Tayo

Table S1. Summarized statistic table of the clinical data controls and demographic profiles of the samples. All samples were from Caucasian descent.

Quality	Mean	Median	Mode	Std. Deviation	Min.	Max.
Age at Death	48.71	51		±13.45	23	70
brain pH	6.81	6.83		±0.16	6.52	7.12
PMI*	22.28	23.75		±8.05	9	40
Gender						
Male			48			
Female			24			

*Post-Mortem Interval

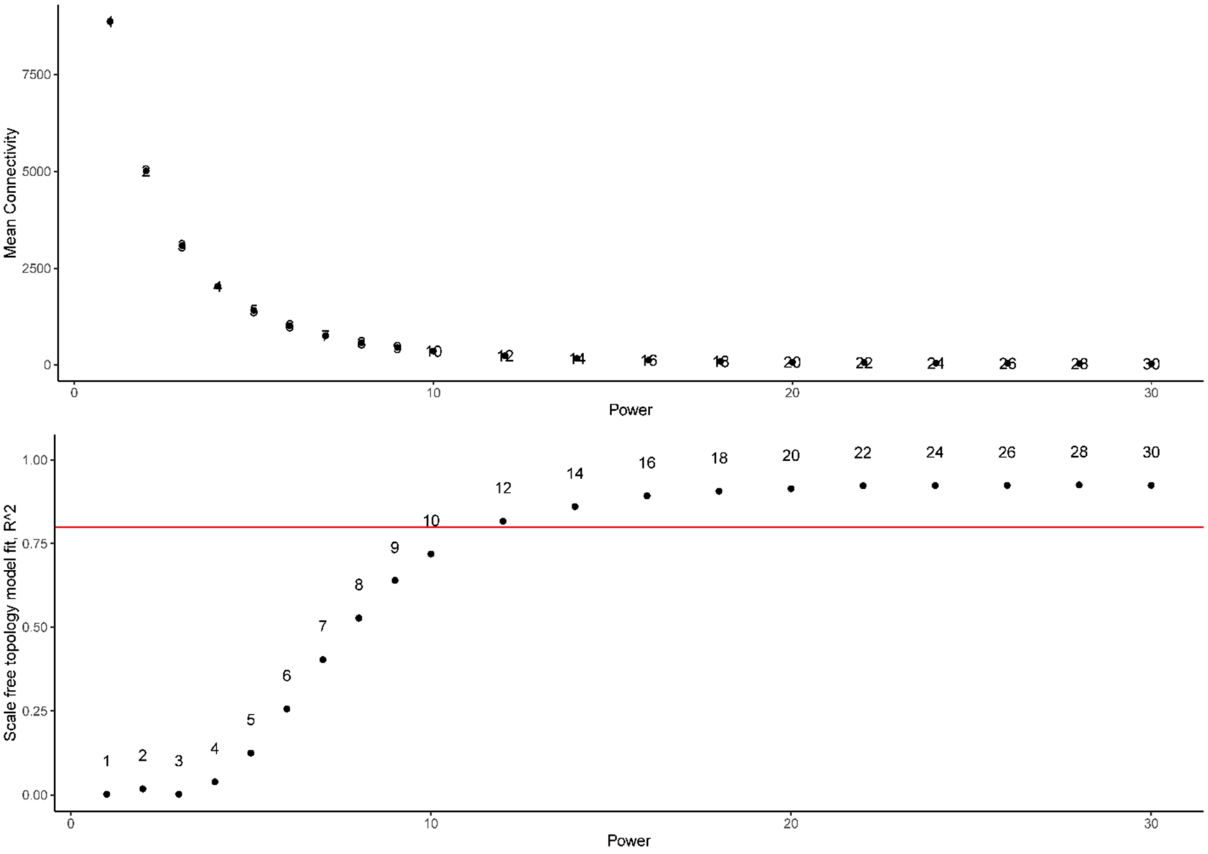

Figure S1. Scale free topology model fit and mean connectivity plots of the pre-processed dataset. Based on the figure, $\beta = 12$ was chosen as the soft-thresholding power in identifying the modules among the three brain regions because it attained the highest mean connectivity among the values that reached the 80% limit.

Table S2. The top 5 clusters from each annotation of the modules identified to have strong expression of gene sets among the three brain regions.

Module	Annotation	Enrichment FDR	Fold Enrichment	ID	Pathway	Genes Involved
blue	BP	1.01E-13	46.974804	GO:0016079	synaptic vesicle exocytosis	SYT1, RIMS1, NAPA, RAB3A, STX1A, SNAP25, SNCA, SYP, VAMP2, SYN1, PIP5K1C
		4.35E-15	41.64	GO:0007269	neurotransmitter secretion	SYN1, SYT1, RIMS1, NAPA, RAB3A, STX1A, SNAP25, SNCA, SYP, CAMK2A, VAMP2, KCNMB4, PIP5K1C
		4.35E-15	41.64	GO:0099643	signal release from synapse	SYN1, SYT1, RIMS1, NAPA, RAB3A, STX1A, SNAP25, SNCA, SYP, CAMK2A, VAMP2, KCNMB4, PIP5K1C
		7.69E-13	29.36	GO:0099504	synaptic vesicle cycle	SYT1, RIMS1, NAPA, RAB3A, STX1A, SNAP25, SNCA, SYN1, RAPGEF4, SYP, VAMP2, PIP5K1C
		1.01E-13	28.64	GO:0006836	neurotransmitter transport	SYN1, SYT1, RIMS1, NAPA, RAB3A, STX1A, SNAP25, SNCA, SYP, CAMK2A, VAMP2, KCNMB4, PIP5K1C
	CC	2.17E-07	486.83	GO:0070032	synaptobrevin 2-SNAP-25-syntaxin-1a-complexin I complex	SNAP25, STX1A, VAMP2
		5.60E-07	365.12	GO:0060199	clathrin-sculpted glutamate transport vesicle	SYT1, RAB3A, VAMP2
		5.60E-07	365.12	GO:0070044	synaptobrevin 2-SNAP-25-syntaxin-1a complex	NAPA, STX1A, VAMP2
		5.60E-07	56.61	GO:0098563	intrinsic component of synaptic vesicle membrane	RAB3A, SYN1, SYT1, STX1A, SYP
		5.60E-07	32.10	GO:0008076	voltage-gated potassium channel complex	KCNMB4, KCNMB2, KCNMA1, STX1A, SNAP25, VAMP2
	MF	1.96E-05	162.276596	GO:0052812	phosphatidylinositol-3,4-bisphosphate 5-kinase activity	PIK3CD, PIP5K1B, PIP5K1C
		2.40E-05	146.048936	GO:0052813	phosphatidylinositol bisphosphate kinase activity	PIK3CD, PIP5K1B, PIP5K1C
		4.69E-05	112.345336	GO:0015269	calcium-activated potassium channel activity	KCNMB4, KCNMA1, KCNMB2
		8.18E-05	91.2805851	GO:0016307	phosphatidylinositol phosphate kinase activity	PIP5K1B, PIK3CD, PIP5K1C
		3.14E-05	48.6829787	GO:0005484	SNAP receptor activity	STX1A, VAMP8, SNAP25, VAMP2
	KEGG	2.33E-11	50.95	hsa04911	Insulin secretion	RAPGEF4, KCNMB4, GNAS, KCNMA1, RAB3A, SNAP25, STX1A, VAMP2, CAMK2A
		1.13E-08	43.69	hsa04721	Synaptic vesicle cycle	RIMS1, RAB3A, SNAP25, STX1A, VAMP2, SYT1, NAPA
		2.28E-09	39.74	hsa04750	Inflammatory mediator reg. of TRP channels	GNAS, IGF1, NTRK1, PIK3CD, PLCG2, MAPK8, MAPK13, CAMK2A

		3.41E-07	34.77	hsa04012	ErbB signaling pathway	EGF, PAK1, PIK3CD, PLCG2, MAPK8, CAMK2A
		6.93E-07	29.81	hsa05231	Choline metabolism in cancer	EGF, PIP5K1C, PIK3CD, MAPK8, RAC3, PIP5K1B
dark green	BP	2.00E-15	114.02	GO:0003351	epithelial cilium movement involved in extracellular fluid movement	SPAG6, CCDC40, SPAG17, DNAAF1, DNAH9, DNAH5, DNAH11, DNAI1, CFAP43
		1.43E-13	109.15	GO:0070286	axonemal dynein complex assembly	ZMYND10, DNAH5, DNAI1, TEKT2, DNAAF1, CCDC40, DNAH2, CFAP100
		3.71E-15	106.42	GO:0006858	extracellular transport	SPAG6, CCDC40, SPAG17, DNAAF1, DNAH9, DNAH5, DNAH11, DNAI1, CFAP43
		1.47E-25	89.62	GO:0035082	axoneme assembly	ZMYND10, DNAH5, SPAG6, RSPH4A, DNAI1, CCDC40, SPAG17, RSPH1, CFAP157, CFAP43, TEKT2, CFAP58, DNAAF1, DRC7, DNAH2, CFAP100
		7.38E-14	77.24	GO:0044458	motile cilium assembly	ZMYND10, SPAG6, CFAP157, CFAP43, CFAP58, SPAG17, DRC7, CCDC40, DNAAF1
	CC	2.34E-08	193.50	GO:0036157	outer dynein arm	DNAH5, DNAI1, CFAP70, DNAH9
		2.46E-11	138.81	GO:0005858	axonemal dynein complex	DNAH5, DNAI1, CFAP70, DNAH9, DNAH2, DNAH3
		8.99E-15	85.52	GO:0030286	dynein complex	DNAH9, DNAH5, DNAH11, DNAI1, DNAH3, DNAH2, CFAP70, DNALI1, DNAH12
		1.32E-27	67.45	GO:0005930	axoneme	DNAH9, DNAH5, SPAG6, DNAH11, RSPH4A, DNAI1, DNAAF1, SPAG17, DNALI1, DNAH2, CFAP43, CCDC40, CFAP70, DRC3, DNAH3, DNAH12, CFAP52, CFAP100
		1.32E-27	66.98	GO:0097014	ciliary plasm	DNAH9, DNAH5, SPAG6, DNAH11, RSPH4A, DNAI1, DNAAF1, SPAG17, DNALI1, DNAH2, CFAP43, CCDC40, CFAP70, DRC3, DNAH3, DNAH12, CFAP52, CFAP100
	MF	7.42E-11	168.04	GO:0008569	minus-end-directed microtubule motor activity	DNAH9, DNAH5, DNAH11, DNAH3, DNAH2, DNAH12
		8.00E-10	106.42	GO:0051959	dynein light intermediate chain binding	DNAH9, DNAH5, DNAH11, DNAH3, DNAH2, DNAH12
		4.69E-03	76.02	GO:0045504	dynein heavy chain binding	DNAI1, DNALI1
		1.21E-02	44.34	GO:0070840	dynein complex binding	DNAAF1, CFAP100
		9.39E-08	43.14	GO:0003777	microtubule motor activity	DNAH9, DNAH5, DNAH11, DNAH3, DNAH2, DNAH12
	KEGG	1.17E-06	13.91	hsa05016	Huntington disease	DNAH2, DNAH5, DNAH9, DNAH12, DNAI1, DNAH3, DNALI1, DNAH11
		2.20E-06	11.69	hsa05014	Amyotrophic lateral sclerosis	DNAH2, DNAH5, DNAH9, DNAH12, DNAI1, DNAH3, DNALI1, DNAH11
		1.10E-05	8.94	hsa05022	Pathways of neurodegeneration-multiple diseases	DNAH2, DNAH5, DNAH9, DNAH12, DNAI1, DNAH3, DNALI1, DNAH11
grey60	BP	1.71E-18	33.05	GO:0002274	myeloid leukocyte activation	TYROBP, TREM2, FCER1G, SYK, CD33, FCGR3A, BTK, PTPN6, DOCK2,

						VSIG4, C1QA, TLR1, TLR6, ITGB2, ITGAM, CD74
		9.44E-15	25.67	GO:0002366	leukocyte activation involved in immune response	TYROBP, CD74, FCER1G, SYK, SPN, FCGR3A, BTK, APBB1IP, TREM2, PTPN6, DOCK2, ITGB2, ITGAM, ITGAL
		8.32E-15	21.68	GO:0002253	activation of immune response	C3, VSIG4, FCER1G, SYK, C3AR1, C1QC, C1QA, BTK, TYROBP, BLNK, TREM2, PTPN6, C1QB, CLEC7A, HCK
		8.33E-19	16.17	GO:0002252	immune effector proc.	TYROBP, CD74, C3, VSIG4, FCER1G, SYK, C1QC, C1QA, SPN, FCGR3A, BTK, APBB1IP, TREM2, PTPN6, DOCK2, C1QB, ITGB2, ITGAM, ITGAL, CLEC7A, HCK
		9.23E-16	15.76	GO:0050778	positive reg. of immune response	CD74, C3, VSIG4, FCER1G, SYK, C3AR1, C1QC, C1QA, BTK, TYROBP, BLNK, TREM2, PTPN6, C1QB, ITGB2, ITGAM, CLEC7A, HCK
	CC	2.35E-07	33.21	GO:0070821	tertiary granule membrane	CD33, ITGAX, FCER1G, ITGB2, CYBB, ITGAM
		3.62E-11	29.33	GO:0098802	plasma membrane signaling receptor complex	ITGAL, ITGAX, ITGAM, TLR1, TLR6, ITGB2, SYK, CSF2RA, APBB1IP, PTPN6
		2.69E-08	22.46	GO:0070820	tertiary granule	CD33, PTPN6, ITGAX, FCER1G, ITGB2, CTSS, CYBB, ITGAM
		2.79E-08	22.14	GO:0042581	specific granule	ITGAL, CD33, PTPN6, DOCK2, ITGB2, CYBB, ITGAM, C3AR1
		3.27E-10	18.03	GO:0030667	secretory granule membrane	ITGAL, TYROBP, CD33, ITGAX, FCGR2A, FCER1G, ITGB2, CYBB, ITGAM, C3AR1, SELL
	MF	1.00E-04	391.13	GO:0030369	ICAM-3 receptor activity	ITGAL, ITGB2
		1.00E-04	391.13	GO:0035663	Toll-like receptor 2 binding	TLR1, TLR6
		1.95E-06	352.02	GO:0001851	complement component C3b binding	VSIG4, ITGB2, ITGAM
		1.16E-05	195.56	GO:0019864	IgG binding	FCER1G, FCGR2A, FCGR3A
		2.65E-05	146.67	GO:0035325	Toll-like receptor binding	TLR1, TLR6, SYK
	KEGG	1.80E-16	68.66	hsa05150	Staphylococcus aureus infection	FCGR2A, FCGR3A, ITGAL, ITGAM, ITGB2, SELPLG, C1QA, C1QB, C1QC, C3, C3AR1
		3.25E-13	62.12	hsa04610	Complement and coagulation cascades	VSIG4, ITGAM, ITGAX, ITGB2, C1QA, C1QB, C1QC, C3, C3AR1
		7.07E-12	61.76	hsa05140	Leishmaniasis	CYBB, FCGR2A, FCGR3A, ITGAM, ITGB2, NCF2, PTPN6, C3
		2.81E-08	46.32	hsa05133	Pertussis	ITGAM, ITGB2, C1QA, C1QB, C1QC, C3
		1.38E-16	42.61	hsa05152	Tuberculosis	TLR6, CTSS, FCER1G, FCGR2A, FCGR3A, ITGAM, ITGAX, ITGB2, CLEC7A, SYK, TLR1, C3, CD74
pink	BP	1.47E-03	311.31	GO:0042414	epinephrine metabolic proc.	TH, PNMT
		1.10E-03	82.40	GO:0001732	formation of cytoplasmic translation initiation complex	EIF3L, EIF3E, EIF3H
		1.10E-03	32.20	GO:0006584	catecholamine metabolic proc.	TH, MAOB, NPY, PNMT

		1.10E-03	32.20	GO:0009712	catechol-containing compound metabolic proc.	TH, MAOB, NPY, PNMT
		2.20E-03	24.26	GO:0050795	reg. of behavior	NPY, HTR1B, HTR1D, PENK
	CC	2.18E-04	107.76	GO:1990907	beta-catenin-TCF complex	TLE4, TCF4, TLE1
		2.18E-04	87.55	GO:0033290	eukaryotic 48S preinitiation complex	EIF3L, EIF3E, EIF3H
		2.18E-04	82.40	GO:0005852	eukaryotic translation initiation factor 3 complex	EIF3L, EIF3E, EIF3H
		2.18E-04	77.83	GO:0016282	eukaryotic 43S preinitiation complex	EIF3H, EIF3L, EIF3E
		2.18E-04	73.73	GO:0070993	translation preinitiation complex	EIF3H, EIF3L, EIF3E
	MF	1.59E-03	233.48	GO:0001515	opioid peptide activity	PDYN, PENK
		3.96E-03	133.42	GO:0031628	opioid receptor binding	PDYN, PENK
		5.77E-03	77.83	GO:0051378	serotonin binding	HTR1B, HTR1D
		6.39E-03	71.84	GO:0043176	amine binding	HTR1B, HTR1D
		5.77E-03	25.016	GO:0015459	potassium channel regulator activity	KCNIP2, KCNAB1, KCNIP1
	KEGG	8.95E-04	38.91	hsa00350	Tyrosine metabolism	MAOB, PNMT, TH
		1.44E-04	38.12	hsa05030	Cocaine addiction	ADCY5, MAOB, PDYN, TH
		6.34E-07	29.18	hsa04726	Serotonergic synapse	ADCY5, GNG7, HTR1B, HTR1D, MAOB, PLCB2, CACNA1S
		2.99E-04	28.74	hsa04927	Cortisol synthesis and secretion	ADCY5, KCNA4, PLCB2, CACNA1S
		3.25E-04	27.07	hsa05031	Amphetamine addiction	ADCY5, MAOB, PDYN, TH
purple	BP	2.06E-02	88.69	GO:0061795	Golgi lumen acidification	ATP6AP1, ATP6V0B
		2.48E-02	76.02	GO:0048388	endosomal lumen acidification	ATP6AP1, ATP6V0B
		6.97E-03	51.50	GO:0007035	vacuolar acidification	ATP6AP1, ATP6V0B, ATP6V0E2
		1.47E-02	31.93	GO:0051452	intracellular pH reduction	ATP6AP1, ATP6V0B, ATP6V0E2
		2.23E-02	24.19	GO:0061951	establishment of protein localization to plasma membrane	ATP6AP1, GGA3, RDX
	CC	4.90E-03	133.03	GO:0008541	proteasome regulatory particle lid subcomplex	PSMD8, PSMD13
		4.90E-03	106.42	GO:0000220	vacuolar proton-transporting V-type ATPase V0 domain	ATP6V0B, ATP6V0E2
		7.81E-03	70.95	GO:0035060	brahma complex	SMARCC2, DPF3
		9.04E-03	62.60	GO:0071565	nBAF complex	SMARCC2, DPF3
		1.51E-02	46.27	GO:0005838	proteasome regulatory particle	PSMD8, PSMD13
	KEGG	2.29E-04	42.57	hsa05110	Vibrio cholerae infection	ATP6V0E2, ARF1, ATP6V0B, ATP6AP1
		2.80E-02	25.96	hsa03440	Homologous recombination	RAD51B, BRCA1
		2.80E-02	23.14	hsa03050	Proteasome	PSMD8, PSMD13
		8.36E-03	22.80	hsa05120	Epithelial cell signaling in Helicobacter pylori infection	ATP6V0E2, ATP6V0B, ATP6AP1
		8.36E-03	20.47	hsa04721	Synaptic vesicle cycle	AP2M1, ATP6V0E2, ATP6V0B
red	BP	5.82E-08	18.31	GO:0046395	carboxylic acid catabolic proc.	HADHA, ALDH6A1, HADHB, GLUD1, ACAA2, GLUD2, ACACB, CPT1A, ALDH4A1, GLUL
		5.93E-08	18.03	GO:0016054	organic acid catabolic proc.	HADHA, ALDH6A1, HADHB, GLUD1, ACAA2, GLUD2, ACACB, CPT1A, ALDH4A1, GLUL

		5.62E-09	15.06	GO:0044282	small molecule catabolic proc.	HADHA, ALDH6A1, HADHB, GLUD1, ACAA2, GLUD2, ALDH3B1, ACACB, CPT1A, ALDH2, ALDH4A1, LRP5, GLUL
		6.21E-08	14.43	GO:0060562	epithelial tube morphogenesis	LRP5, ABL1, TGFB1, GNA13, CXCR4, SDC4, SMO, NOTCH2, NOTCH1, BCL2, DAG1
		5.82E-08	11.04	GO:0002009	morphogenesis of an epithelium	LRP5, DAG1, ABL1, TGFB1, GNA13, CXCR4, SDC4, SMO, NOTCH2, EGFR, NOTCH1, BCL2, GJA1
	CC	1.21E-03	37.86	GO:0005834	heterotrimeric G-protein complex	GNA13, GNA12, GNG12
		1.34E-03	35.02	GO:1905360	GTPase complex	GNA13, GNA12, GNG12
		1.21E-03	12.16	GO:0001726	ruffle	EZR, EGFR, MTM1, ABL1, TLN1
		8.95E-08	10.78	GO:0005759	mitochondrial matrix	HADHA, ACSS3, HADHB, ACSS1, ALDH2, ALDH4A1, GLUD2, ALDH6A1, PYCR2, GLUD1, ALDH7A1, ACAA2
		1.45E-03	10.52	GO:0005741	mitochondrial outer membrane	BCL2, HADHB, CPT1A, MAOA, ACACB
	MF	2.95E-04	311.31	GO:0004352	glutamate dehydrogenase (NAD+) activity	GLUD1, GLUD2
		2.95E-04	311.31	GO:0004353	glutamate dehydrogenase [NAD(P)+] activity	GLUD1, GLUD2
		2.95E-04	311.31	GO:0004354	glutamate dehydrogenase (NADP+) activity	GLUD1, GLUD2
		2.95E-04	311.31	GO:0016639	oxidoreductase activity acting on the CH-NH2 group of donors NAD or N	GLUD1, GLUD2
		1.21E-05	233.48	GO:0003985	acetyl-CoA C-acetyltransferase activity	ACAA2, HADHB, HADHA
	KEGG	2.74E-08	106.18	hsa00340	Histidine metabolism	ALDH2, ALDH3B1, ALDH9A1, MAOA, ALDH7A1
		2.54E-09	90.38	hsa00410	beta-Alanine metabolism	ALDH2, ALDH3B1, ALDH9A1, HADHA, ALDH6A1, ALDH7A1
		6.30E-05	82.40	hsa00910	Nitrogen metabolism	GLUD1, GLUD2, GLUL
		1.44E-07	72.96	hsa00640	Propanoate metabolism	HADHA, ACACB, ALDH6A1, ACSS3, ACSS1
		9.21E-10	65.37	hsa00330	Arginine and proline metabolism	ALDH2, ALDH9A1, GATM, PYCR2, MAOA, ALDH7A1, ALDH4A1
turquoise	BP	1.78E-06	153.05	GO:0006122	mitochondrial electron transport ubiquinol to cytochrome c	UQCRB, UQCRFS1, UQCRC1, UQCRC2
		5.76E-07	32.24	GO:0019646	aerobic electron transport chain	SDHA, UQCRB, NDUFS2, UQCRFS1, UQCRC1, SDHB, UQCRC2
		7.34E-10	30.52	GO:0006119	oxidative phosphorylation	SDHA, UQCRB, NDUFS2, UQCRFS1, UQCRC1, SDHB, UQCRC2, ATP5F1A, ATP5F1C, VCP
		7.41E-07	30.02	GO:0042773	ATP synthesis coupled electron transport	SDHA, UQCRB, NDUFS2, UQCRFS1, UQCRC1, SDHB, UQCRC2
		1.78E-06	25.42	GO:0022904	respiratory electron transport chain	SDHA, SDHB, UQCRB, NDUFS2, UQCRFS1, UQCRC1, UQCRC2
	CC	2.59E-07	142.12	GO:0005750	mitochondrial respiratory chain complex III	UQCRB, UQCRFS1, UQCRC2, UQCRC1

	3.18E-07	132.64	GO:0005665	RNA polymerase II core complex	POLR2B, POLR2F, POLR2D, POLR2K
	6.75E-08	31.65	GO:0098803	respiratory chain complex	SDHA, UQCRB, NDUFS2, UQCRFS1, SDHB, UQCRC2, UQCRC1
	7.34E-08	30.54	GO:0005746	mitochondrial respirasome	SDHA, UQCRB, NDUFS2, UQCRFS1, SDHB, UQCRC2, UQCRC1
	1.04E-07	28.78	GO:0070469	respirasome	SDHA, UQCRB, NDUFS2, UQCRFS1, SDHB, UQCRC2, UQCRC1
MF	1.94E-04	331.61	GO:0008177	succinate dehydrogenase (ubiquinone) activity	SDHA, SDHB
	3.31E-04	248.71	GO:0000104	succinate dehydrogenase activity	SDHA, SDHB
	8.75E-07	198.97	GO:0043560	insulin receptor substrate binding	PIK3CB, PIK3CA, PIK3R1, GRB2
	1.63E-04	18.29	GO:0009055	electron transfer activity	SDHA, SDHB, NDUFS2, UQCRFS1, UQCRC1
	3.17E-06	13.17	GO:0031625	ubiquitin protein ligase binding	CUL3, CUL1, PRKACB, BECN1, UQCRC1, GSK3B, HSPA8, NDUFS2, VCP
KEGG	6.93E-12	64.18	hsa04213	Longevity regulating pathway-multiple species	MTOR, HSPA8, KRAS, NRAS, PIK3CA, PIK3CB, PIK3R1, PRKACB
	4.81E-11	50.37	hsa01521	EGFR tyrosine kinase inhibitor resistance	MTOR, GRB2, GSK3B, KRAS, NRAS, PIK3CA, PIK3CB, PIK3R1
	7.17E-11	47.37	hsa04012	ErbB signaling pathway	MTOR, GRB2, GSK3B, KRAS, NRAS, PIK3CA, PIK3CB, PIK3R1
	7.74E-11	46.27	hsa05210	Colorectal cancer	MTOR, GRB2, GSK3B, KRAS, NRAS, PIK3CA, PIK3CB, PIK3R1
	6.45E-13	41.45	hsa04935	Growth hormone synthesis secretion and action	MTOR, GNAI1, GRB2, GSK3B, KRAS, NRAS, PIK3CA, PIK3CB, PIK3R1, PRKACB

Table S3. List of genes found to have significant association with BPD. Those which showed presence of SNPs were also recorded. Moreover, degree corresponds to the number of first-degree neighbors adjacent to the gene, which was obtained from the PPI network constructed from the expression profiles of BPD patients.

Sample	Gene	GDA	Degree	SNPs	Consequence
nAcc	*DRD2*	0.400	31	rs1801028	missense variant
	PPP1R1B	0.350	9		
	CHRNA2	0.300	3		
	GFRA2	0.100	4	rs7833426	intron variant
	DCBLD1	0.100		rs62433108	intron variant
	PENK	0.010	6		
	NOS1AP	0.010	3		
	PART1	0.010			
	NRN1	0.010			
AnCg	*GRIK4*	0.350	4		
	ST8SIA2	0.350	3	rs4777989	intron variant
	FABP7	0.320			
	TSHZ1	0.310			

	ADRA1A	0.300	6		
	ADAMTS16	0.100		rs16875288	intron variant
	HTR7	0.010	15		
	PCDH17	0.010			
	PNPLA3	0.010	9		
DLPFC	*IGFBP2*	0.350	4		
	SLC17A6	0.310	4		
	FOXO3	0.310	67	rs1536057<br>rs1935952<br>rs2802292	intron variant
	ITGA9	0.310	7	rs166508	intron variant
	CARTPT	0.310	1		
	PREP	0.310	6		
	NEFM	0.300	13		
	CUBN	0.100	3	rs7904579	intron variant
	PLCB4	0.100	30	rs2299682	intron variant
	NOS1	0.060	47		
	NOS2	0.050	16		
	FGF9	0.030	13		
	RORB	0.010	8	rs1327836	intron variant
	SGCG	0.010	10		
	ZNF365	0.010	1		

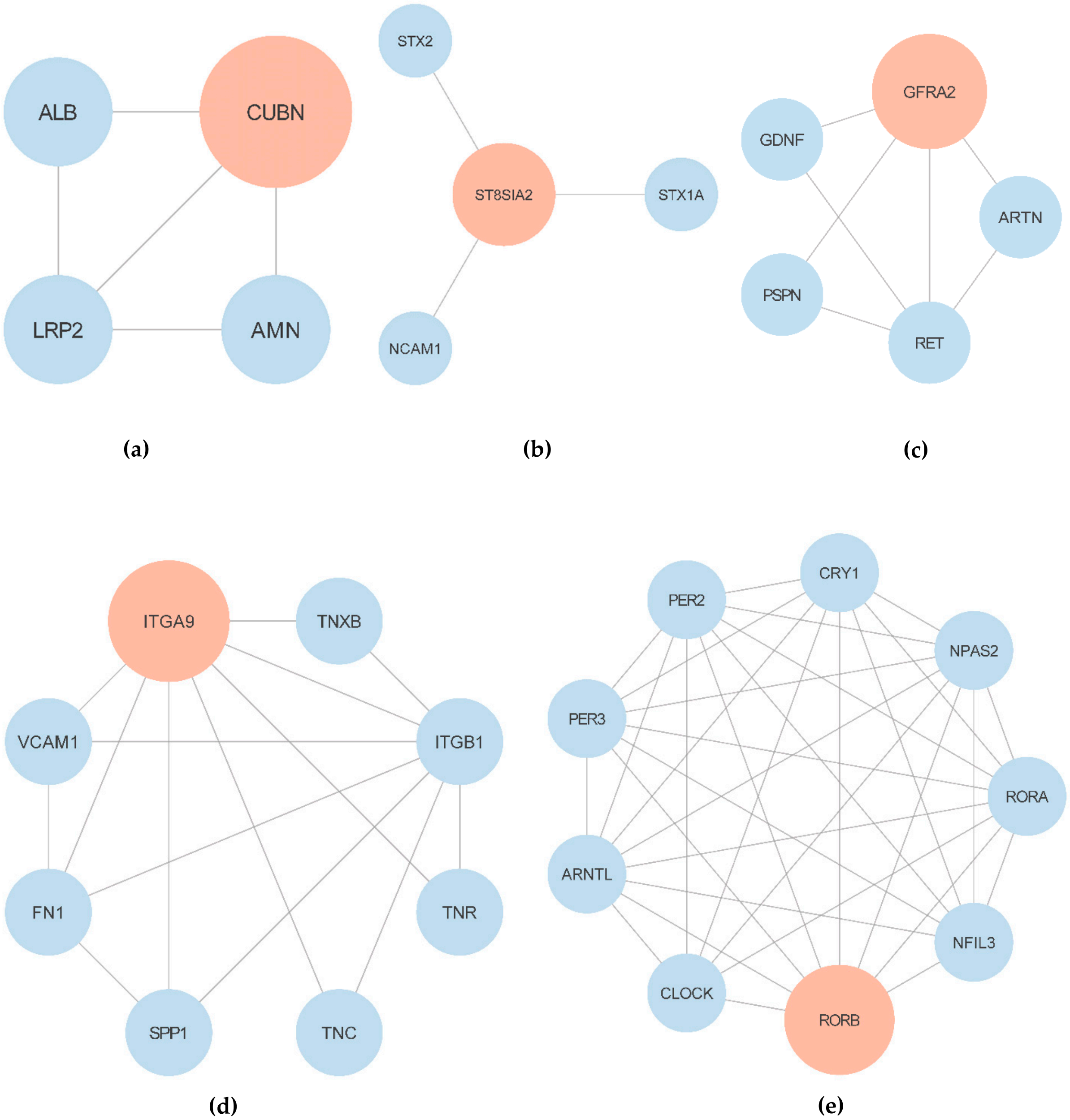
ALB
CUBN
LRP2
AMN
(a)
STX2
ST8SIA2
STX1A
NCAM1
(b)
GFRA2
GDNF
ARTN
PSPN
RET
(c)
ITGA9
TNXB
VCAM1
ITGB1
FN1
TNR
SPP1
TNC
(d)
CRY1
PER2
NPAS2
PER3
RORA
ARNTL
NFIL3
CLOCK
RORB
(e)

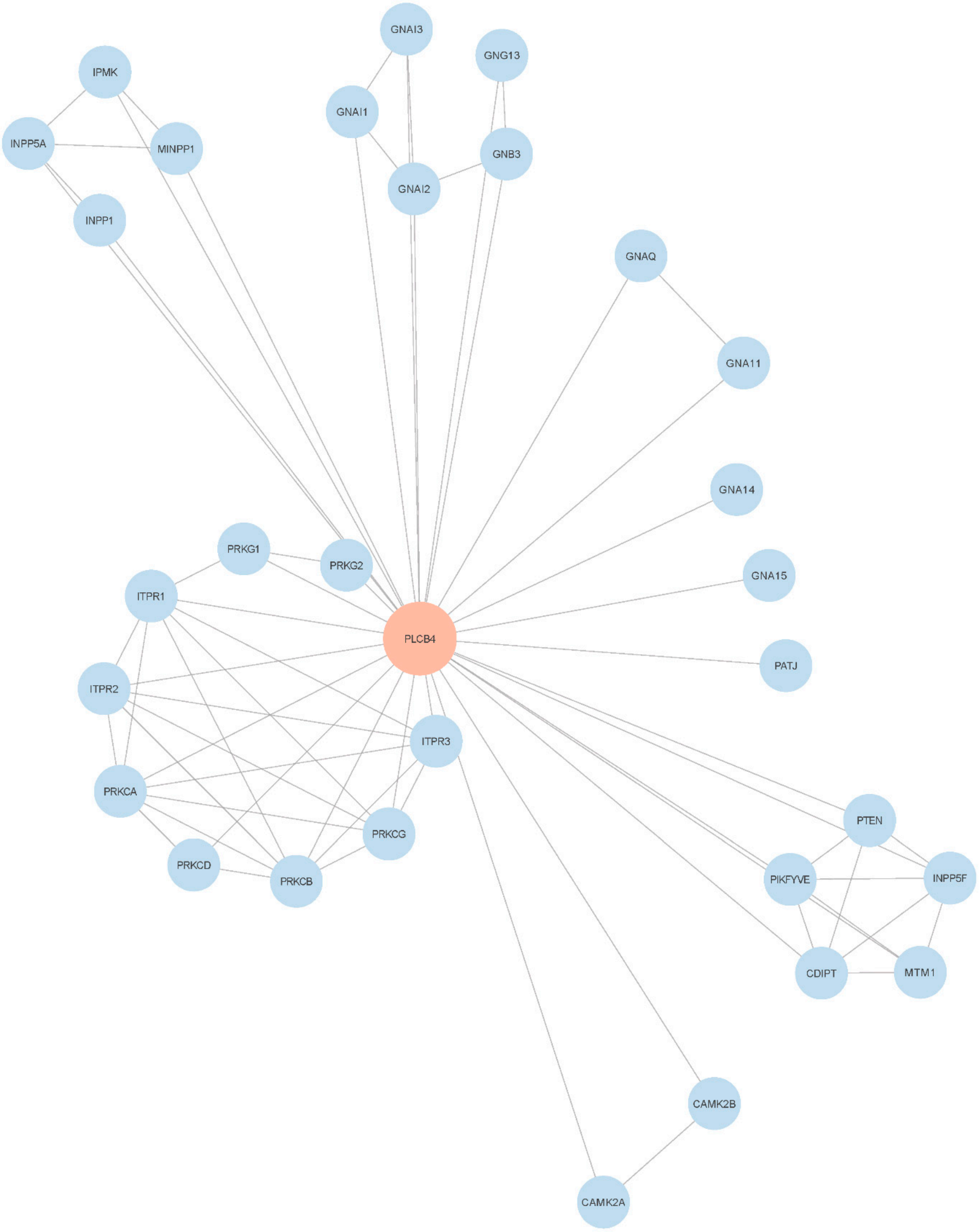
GNAI3
GNG13
IPMK
GNAI1
INPP5A
MINPP1
GNB3
GNAI2
INPP1
GNAQ
GNA11
GNA14
PRKG1
PRKG2
GNA15
ITPR1
PLCB4
PATJ
ITPR2
ITPR3
PRKCA
PRKCG
PTEN
PRKCD
PRKCB
PIKFYVE
INPP5F
CDIPT
MTM1
CAMK2B
CAMK2A

(f)

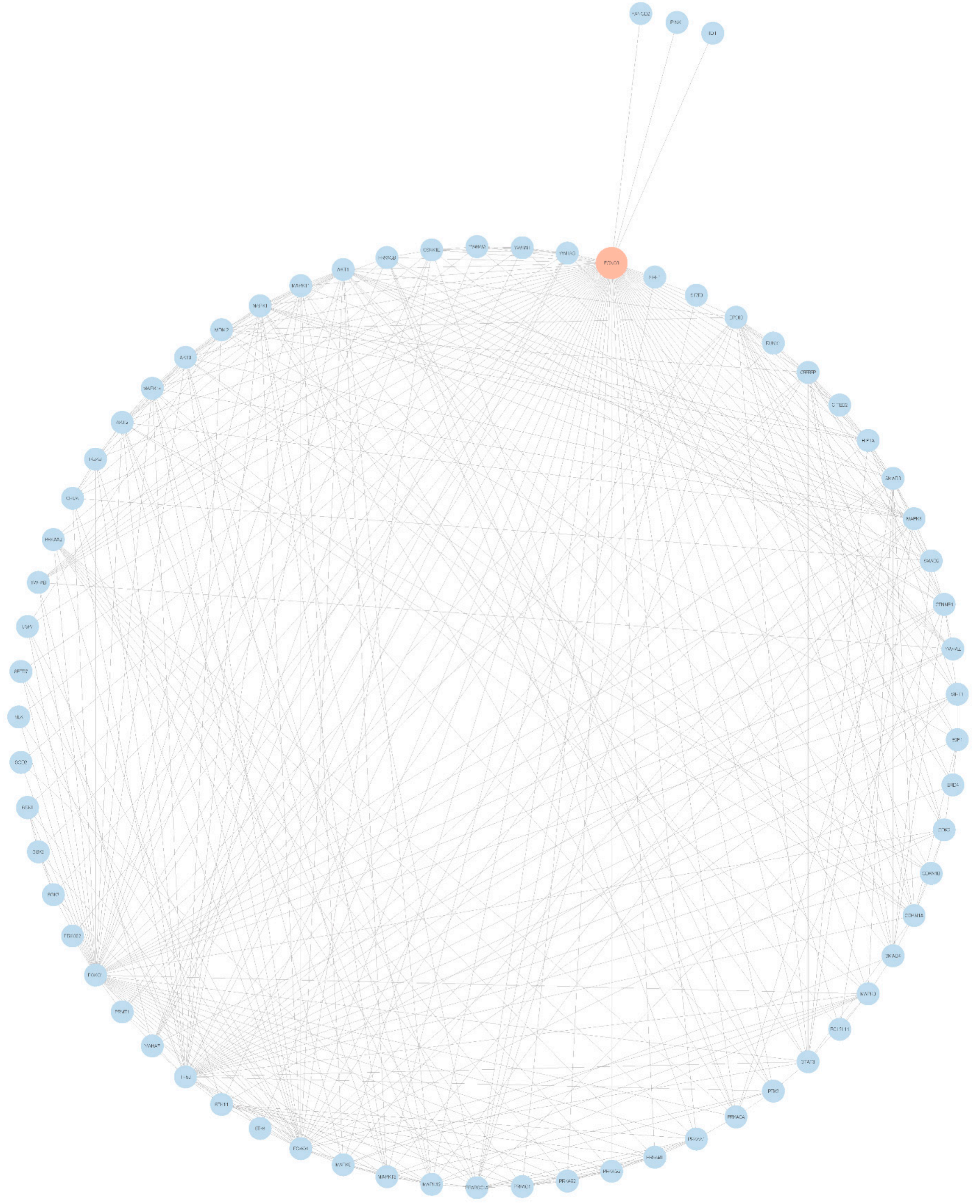

(g)

Figure S2. Modified PPI network of the genes that were identified to have disease-associated variants among the three brain regions: (**a**) *CUBN*; (**b**) *ST8SIA1*; (**c**) *GFRA2*; (**d**) *ITGA9*; (**e**) *RORB*; (**f**) *PLCB4*; (**g**) *FOXO3*.
